# Sarcoma of the Superior Maxilla in a Child of Eight—Removal

**Published:** 1884-06

**Authors:** 


### Sarcoma of the Superior Maxilla in a Child of Eight—Removal.

Dr. L. W. Marshall reports (*British Medical Journal*) a case occurring in the Nottingham Hospital. When admitted the growth was of one year's standing and had attained the size of a walnut in the right superior maxilla. There was no pain, tenderness, discoloration, or adhesion to the tumor. Operation was postponed. Six months later the case returned, the growth having steadily progressed. The bicuspid and first molar teeth had fallen out and the swelling was encroaching on the mouth, nose, and orbit. It was removed by Heath's incision. The orbital plate and posterior part of the maxilla were left, scraped, and washed with a chloride of zinc solution, as the tumor seemed to spring from the lining membrane of the antrum.

The patient did well and was discharged from the hospital in eight weeks. At the end of a year there was no sign of return, and the patient was but slightly disfigured.

The portion removed consisted of the greater part of the maxilla and a part of the palate bones. The growth had filled the antrum; microscopically, it was composed of spindle-shaped cells, connective tissue, and free nuclei.

